# Obesity-related CpG Methylation (cg07814318) of Kruppel-like Factor-13 (*KLF13*) Gene with Childhood Obesity and its *cis*-Methylation Quantitative Loci

**DOI:** 10.1038/srep45368

**Published:** 2017-05-16

**Authors:** In-Uk Koh, Hye-Ja Lee, Joo-Yeon Hwang, Nak-Hyun Choi, Suman Lee

**Affiliations:** 1 Division of Structural and Functional Genomics, Center for Genome Science, National Institute of Health, Chungcheongbuk-do, 363-951, Republic of Korea; 2 Division of Metabolic Diseases, Center for Biomedical Sciences, National Institute of Health, Chungcheongbuk-do, 361-951, Republic of Korea

## Abstract

The cg07814318 hypermethylation of Kruppel-like factor 13 (*KLF13*) gene has been reported for its relevancy with Body Mass Index (BMI) from European origin. We explored the cg07814318 methylation and its *cis*-meQTL (*cis*-methylation quantitative loci) of *KLF13* from a childhood obesity cohort. The cg07814318 methylation in blood was significantly associated with obesity and correlated with several obesity-related physical and biochemical traits. We examined the same loci from purified three human cell types (n = 47), i.e., pre-adipocytes, adipocytes and islets. The cg07814318 methylation pattern in pre-adipocytes and islets were significant higher in cells from subjects with a higher BMI compared with control subjects. By exome sequencing of *KLF13* gene in blood with the same cohort, we found nine SNPs (single nucleotide polymorphisms) within its gene body, and two SNPs (rs11537749 and rs12595641) were as *cis*-meQTL of cg07814318. There was the 2.01% methylation change of cg07814318 between homozygous dominant and recessive genotypes, especially, in rs12595641. The sequencing variations within *KLF13* genes could drive dynamic modifications of obesity-related CpG methylation. Differential DNA methylation patterns in the *KLF13* gene determined from separate blood samples showed that this criterion could be used as a surrogate for representing overall epigenetic changes in cells related to obesity.

Obesity is a condition with excessive body fat accumulation resulting from a combination of genetic and environmental contributing factors. Childhood obesity is defined as a Body Mass Index (BMI) at or above the 95^th^ percentile for children and teens of the same age and sex. Childhood obesity is the complex disease itself, and increases the future risk of many chronic diseases, and predisposes cardiovascular diseases, hypertension and type 2 diabetes^1^,^2^.

Genome Wide Association Studies (GWASs) have identified hundreds of genetic variants associated with complex human diseases and traits and provided valuable insights into their genetic architectures related to diseases^3^. Obesity is heritable, and genetic association studies have shown statistically significant genomic loci associated with its susceptibility^4^. More precisely, several GWASs with obesity have confirmed that the statistically associated loci, transcription factor 7-like 2 (*TCF7L2*) and fat mass and obesity-associated protein (*FTO*), may provide novel insight into the pathophysiology of obesity^4^. GWAS has successfully been used to elucidate the genetic variants that influence type 2 diabetes (T2D) and BMI. A GWAS study using up to 339,224 individuals identifies 97 BMI-associated loci, and genome-wide estimates, suggesting that this common variation accounts for >20% of BMI variation^5^. Therefore, most variants identified confer minor risk and explain only a small proportion of BMI variation or obesity^6^.

Previous epigenetic studies have suggested that DNA methylation could contribute to explaining the missing heritability of obesity^6^. The epigenetic regulation of genes might play an important role in the susceptibility to obesity. An Epigenome-Wide Association Study (EWAS) focused on obesity showed that hypermethylation of differential methylation positions (DMPs) in intron 1 of Hypoxia-inducible factor–3 alpha (*HIF3A*) and cg07814318 in intron 1 of Kruppel-like factor 13 (*KLF13*) are associated with high BMI from 479 individuals of European origin discovery cohort^7^. Subsequent studies with *HIF3A* suggested that alterations of DNA methylations in *HIF3A* and their cis-meQTL (rs8102595 and rs3826795) were associated with obesity and related traits^7^,^8^,^9^,^10^. Dick *et al*. reported that the differential methylation of *KLF13* on chromosome 17, in addition to *HIF3A*, had strong association with BMI in their discovery cohort, but was not replicated. Here, we investigated BMI-related differential methylation position (DMP), cg07814318, of *KLF13* in blood by pyrosequencing in relation to extreme childhood obesity.

*KLF13* is a member of a family of zinc finger-containing transcription factors, which positively regulates expression of the chemokine, Regulated on Activation, Normal T Cell Expressed and Secreted (*RANTES*), 3–5 days after activation of T cells^11^. *KLF13* gene expression was also regulated by glucocorticoid in cardiomyocytes of the mouse heart^12^. In an obesity-related study of *KLF13*, the expression of KLF13 was markedly up-regulated during the early stage of porcine adipocyte differentiation, which was followed by expression of *PPARγ*^13^.

We also investigated cg07814318 methylation loci from three different purified cell types (pre-adipocytes, adipocytes, and islets) depending on BMI of cell donor subjects for its relevance of surrogate blood cg07814318 methylation. We further investigated the genetic base of childhood obesity by exome sequencing of KLF13 from same cohort (n = 692). There is the interplay between genetic and epigenetic variations, and so CpG methylation can be measured as methylation quantitative trait loci (meQTL). We investigated meQTL of target cg07814318 methylation based on exome sequencing of *KLF13* gene.

Here, we revealed the crosstalk of genetic and epigenetic events of *KLF13* gene in extreme childhood obesity. This study could suggest the integrated effect of genetic and epigenetic changes in childhood obesity.

## Results

### General characteristics of the obese children and controls

The general characteristics of obese children and age- and sex-matched controls are summarized in Table 1. Extreme childhood obese cases (n = 305) with an average BMI of 31.71 ± 3.94 and lean controls (n = 387) with an average BMI of 19.44 ± 1.34 were categorized in KoCAS. There was no significant difference between the two groups in age or gender (P > 0.05). The averages of waist-hip-ratio (WHR), fasting glucose levels (FPG), hepatotoxicity measurements, AST (Aspartate transaminase) and ALT (Alanine aminotransferase), lipid levels, TC (Total Cholesterol), TG (Triglyceride) and HDL (High-density lipoproteins -Cholesterol) were also listed with significant differences between cases and controls in Table 1.

### Target DMPs analysis with the controls and cases of childhood obesity

We examined two differentially methylated positions (DMPs) in an intron of *KLF13* by pyrosequencing. Twenty one samples were failed for the experiment, and finally the controls (n = 374) and the cases (n = 297) subjects were included for pyrosequencing analysis. The details for subject’s criteria are described in the Experimental Procedures section. Two investigated DMPs (cg07814318 and cg31624588) were located in intron 1 of the *KLF13* gene.

Target pyrosequencing of DMPs combines a simple reaction protocol with reproducible and accurate measures of the degree of methylation. We determined two CpG sites (cg07814318 and cg31624588) by pyrosequencing. cg31624588 is located 4 bases after cg07814318, which named by chromosome base pair location (hg19). The genomic position and gene positions of the two target DMPs analyzed via pyrosequencing are described in Supplementary Figure 1. Tested CpG sites were available for pyrosequencing by PCR reaction. One set of primer sequences and PCR conditions are shown in Supplementary Figure 1. An average distribution by pyrosequencing analysis had shown from all childhood obesity (n = 671) in Table 2. The only cg07814318 were, especially, differentially methylated between the controls and the cases (Table 2). The methylation of cg07814318 had increased by 1.4% in cases than the control, and significant associations with childhood obesity (*P* values = 2.305e-8).

### Target DMP of *KLF13* gene associated with other obesity-related traits

To investigate the potential biological significance of the DMPs, we interrogated the DMP (cg07814318) with BMI, waist-hip-ratio (WHR), fasting glucose levels (FPG), hepatotoxicity traits (AST: Aspartate transaminase and ALT: Alanine aminotransferase), lipid levels (TC: Total Cholesterol, TG: Triglyceride and HDL: High-density lipoproteins -Cholesterol) (n = 683). The correlation with *Beta* and *P* values between the methylation of cg07814318 and eight obesity related traits (BMI, WHR, FPG, AST, ALT, TC, TG and HDL) at total and control cohort were shown in Table 3. cg07814318 methylation was significantly correlated with BMI (*Beta* = 0.4048, *P* = 1e-06) in total cohort with sex and age as covariates (n = 671). For every 1% increase in cg07814318 methylation of islet cells, BMI of donors was increased by 0.4 kg/m^2^. The cg07814318 methylations with 8 obesity related traits showed all positive correlations, except FPG. WHR (*Beta* = 0.0051, *P* = 5.801e-14), AST (*Beta* = 0.2456, *P* = 1.495e-08), ALT (*Beta* = 0.6433, *P* = 1.915e-06), TC (*Beta* = 0.2027, *P* = 0.0015) and high-density lipoproteins-cholesterol (*Beta* = −0.45350, *P* = 9.605e-05) had all positive correlations, but FPG (*Beta* = −0.1869, *P* = 0.0002) has negative correlation. In control cohort with normal BMI (n = 374), the correlation of BMI and cg07814318 was diminished. The correlations of cg07814318 with other traits were all maintained with different *Beta* values, and the significance of correlation with WHR (*Beta* = 0.0002, *P* = 1.196e-14) and AST (*Beta* = 0.059, *P* = 1.279e-11) are strongly maintained. The correlation direction for FPG was also negatively maintained in control subjects.

### cg07814318 methylation of pre-adipocytes, adipocytes and islets depending on BMI of donors

The cg07814318 methylation was also investigated for its concordance with the same locus in DNA isolated from human cells for its relation with blood DNA methylation. We determined cg07814318 methylation via the Illumina Infinium HumanMethylation450 BeadChip from 8 pre-adipocytes, 22 adipocytes purified from abdominal fat tissues and 17 islets from a normal pancreas. The averages of CpG methylations of each cell group were shown by controls and cases depending on BMI of donors whom the cells come from (Supplementary Table 1).

cg07814318 methylations were higher in cases in total cells with higher BMI (>29) donors (n = 47). There was a significance in pre-adipocytes (n = 8, *P* = 0.006) and in islets (n = 17, *P* = 0.003). In pre-adipocytes and islets, cg07814318 methylation from obese subjects was about 4–5% higher on the average significantly, but, in adipocyte, it was similar between the controls and cases (Supplementary Table 1).

**Figure 1:**
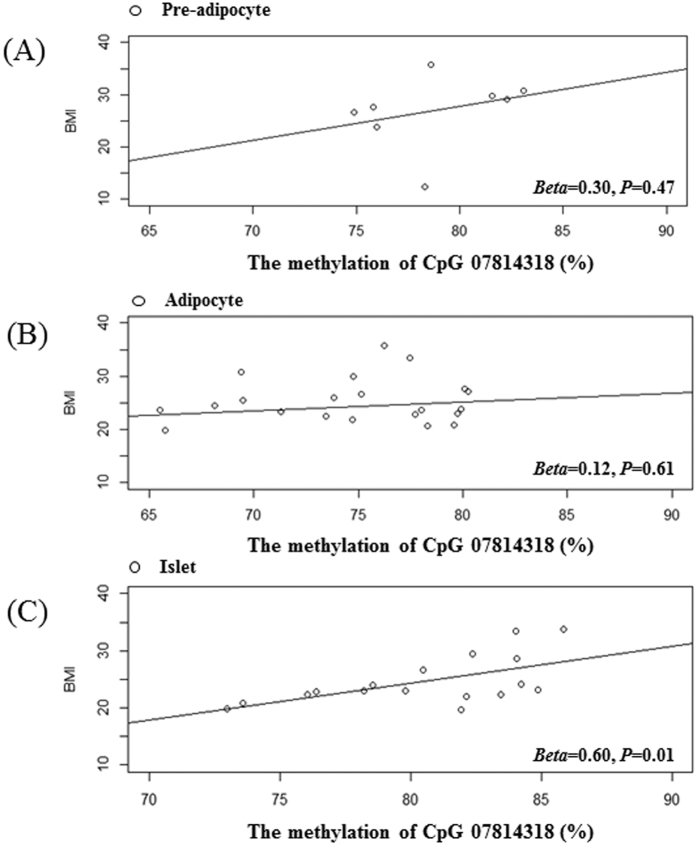
The correlation graph of cg07814318 methylation in three human cell types with BMIs of donors. (**A**) The methylation percentage of cg07814318 in 8 pre-adipocytes with BMI of donors, (**B**) The methylation percentage of cg07814318 in 22 adipocytes with BMIs of donors, (**C**) The methylation percentage of cg07814318 in 17 islets with BMIs of donors.

The correlations of cg07814318 and BMI were graphed in pre-adipocytes (Fig. 1A), adipocytes (Fig. 1B) and islets (Fig. 1C). For every 1% increase in methylation *Beta* value at cg07814318 of pre-adipocytes (n = 8), adipocytes (n = 22) and islet cells (n = 17), BMI of donors in each cell group was 0.30, 0.12 and 0.60 kg/m^2^ increased. The only BMI of islet donors was significantly correlated with cg07814318 methylation (*P* = 0.01). In pre-adipocytes, the BMI of cell donors was positively correlated with cg07814318 methylation (*Beta* = 0.3), but not significant (*P* = 0.47).

### Genetic association study of exome variants with childhood obesity

**Figure 2:**
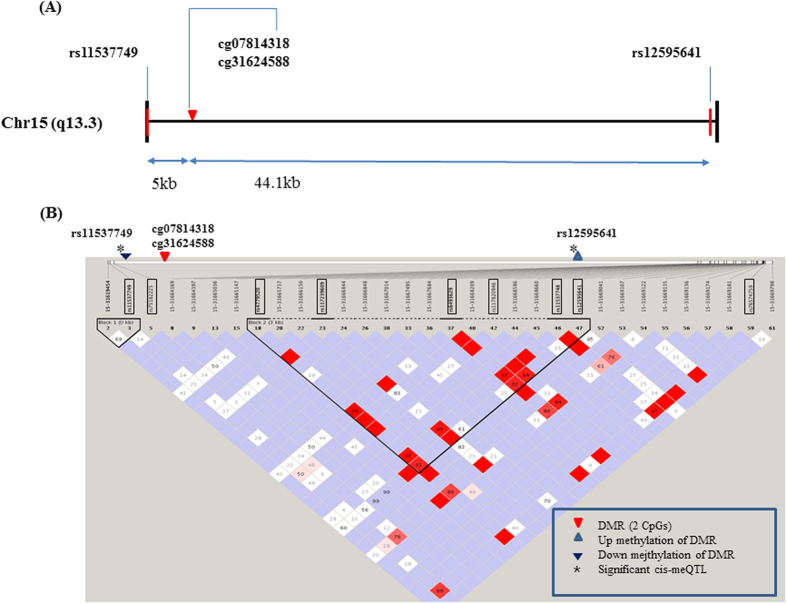
Schematic gene structure of KLF13 locus with DMPs and SNPs. (**A**) The BMI-related DMPs (cg07814318 and cg31624588) in intron 1 is indicated by a red triangle. (**B**) Two SNPs (rs11537749 and rs12595641) with significant correlations with 2 DMPs are indicated on LD block, as is the direction of methylation change (up-regulation, up-arrow; downregulation, down-arrow).

We performed exome sequencing of 692 blood samples. The methodological details are described in the Experimental Procedures section. We found 9 sequencing variants (rs11537749, rs75182225, rs4779520, rs117239609, rs6493629, rs117820946, rs11537748, rs12595641, rs76574759) of *KLF13* in exons and introns (Table 4). One SNP (rs11537749) is a synonymous polymorphic variation and located at the upstream of two BMI-related DMPs, and other 8 SNPs at the downstream (Fig. 2A). From the genetic association study with 692 subjects (387 controls and 305 cases), all nine SNPs showed no significant association with childhood obesity. Linkage Disequilibrium block (LD) was shown in Fig. 2B. In intron 1 of *KLF13* gene, there is one LD block.

**Figure 3:**
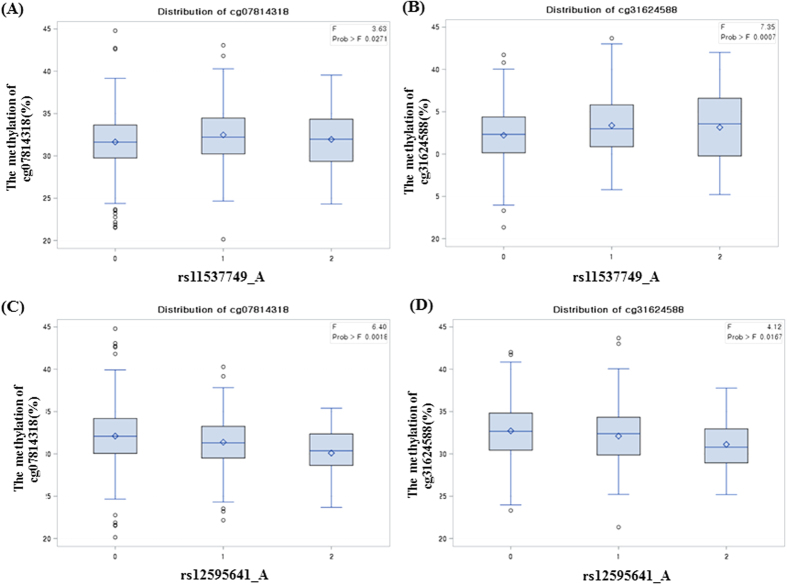
The distribution of cg07814318 and cg31624588 methylations by the genotype of rs11537749 and rs12595641. A diagram of the distribution of DNA methylation percentages of 2 CpG sites (cg07814318 and cg31624588) depending on the genotypes of rs11537749 and rs12595641, 0 (homozygous for dominant allele), 1 (heterozygous), 2 (homozygous for recessive allele). (**A**) The methylation percentage of cg07814318, (**B**) cg31624588 depending on the rs11537749 genotypes; (**C**) The methylation percentage of cg07814318, (**D**) cg31624588 depending on the rs12595641 genotypes. The distribution of two CpG methylations by the genotype of rs11537749 and rs12595641.

To investigate genetic and epigenetic integration effects, we further performed a *cis*-meQTL analysis with nine SNPs and two CpG sites (cg07814318 and cg31624588). We found two SNPs, rs11537749, and rs12595641 had correlations with two CpG sites. rs11537749 is located 5 kb upstream of DMPs in exon 1 of KLF13, and rs12595641 is located 44.1 kb downstream of DMPs (Fig. 2A). SNPs that were newly identified by *cis*-meQTL analysis (rs11537749 and rs12595641) showed the weak linkage disequilibrium (LD) (Fig. 2B). Two DMPs were altered in the same direction within each genotype of SNP. The methylation changes of 2 CpG sites from homologous dominant genotype to homologous recessive genotypes were the negative direction (hypomethylation) in rs12595641, as were the positive direction (hypermethylation) in rs11537749 (Fig. 2). The relations of the methylation of two DMPs (cg07814318 and cg31624588) with the genotypes of two SNPs (rs11537749 and rs12595641) were graphed in Fig. 3. There were significant changes of two CpG sites (cg07814318 and cg31624588) with positive (rs11537749, Fig. 3A,B) or negative (rs12595641, Fig. 2C,D) direction, respectively. The relationship of the methylation of DMPs with the genotypes of rs11537749 and rs12595641, (0: homozygous for the dominant allele, 1: heterozygous, 2: homozygous for the recessive allele) are graphed in Fig. 2. The homozygous recessive and hetero genotype of rs11537749 had hypermethylated than the homozygous dominant genotype at all two CpG sites. In the case of cg07814318, the methylation change between homozygous dominant and recessive genotypes caused a difference of 0.3% in rs11537749 and −2.01% in rs12595641 depending on the genotypes (Table 5).

In the case of cg31624588, the methylation change between homozygous dominant and recessive genotypes caused a difference of 0.93% in rs11537749 and −1.6% in rs12595641 depending on the genotypes (Table 5). The methylation percentages of hetero genotypes of rs12595641 were always in the middle values between major and minor genotype values, but not rs11537749 (Table 5).

## Discussion

An increased food intake and decreased energy expenditure in childhoods may drive the increasing occurrence of obesity greatly during the past three decades^2^. Here, we found obesity-related cg07814318 methylation in the blood of childhood obesity. Our data support the previous reports showing cg07814318 methylation change of *KLF13* in BMI of European adult obesity^7^. The strong correlation of *KLF13* methylation with 8 obesity-related traits (BMI, WHR, FPG, AST, ALT, TC, TG and HDL) suggested that environmental factors may directly drive the epigenetic perturbation of certain chromosomal loci^7^,^9^,^10^. Strong correlations of cg07814318 methylation with obesity-related traits suggested epigenetic mechanisms may explain a dynamic role in excessive fat deposition and weight reduction affecting weight homeostasis. Previously, Wang *et al*. also showed that *HIF3A* DNA methylation in blood is associated with childhood obesity, and has a BMI-independent association with alanine aminotransferase level (ALT)^10^. In addition to *HIF3A* DNA methylation, this study is the first study to validate the association of cg07814318 methylation in childhood obesity.

We also found the significant concordance of obesity-related cg07814318 methylation in blood and two human cells, pre-adipocytes and islets. Differential DNA methylation patterns in the *KLF13* gene of blood samples showed that this criterion could be used as a surrogate for representing epigenetic changes in cells related to obesity.

In pancreatic islets, the significant differential cg07814318 hyper-methylations at donors with higher BMI imply that DMP may be biologically relevant to obesity. The epigenetic perturbation of *KLF13* with BMI was increased at cg07814318 in islet compared to circulating blood (Δ4.64% to Δ1.4%). In pre-adipocytes, the cg07814318 methylations were also increased at high BMI donors (Δ4.38%) like islet types, but adipocytes had similar methylation pattern between normal and cases.

Knockout (KO) mouse study of *KLF13* showed that activated T lymphocytes from KO mice had decreased expression of chemokine RANTES which has been implicated in many inflammatory diseases, including insulin resistance and obesity^14^. Klf13(−/−) mice also exhibited enlarged thymi and spleens. Inflammation is closely linked to obese states with the development of metabolic dysfunction, which suggest an implication of *KLF13* in obesity^15^. In addition to inflammation*, KLF13* was also reported as a pro-adipogenic transcription factor, through transactivating PPARγ a key regulator of adipocyte differentiation^13^. This differential cg07814318 methylation of *KLF13* with pre-adipocytes related to obesity may imply its early epigenetic role in differentiation or proliferation of pre-adipocytes.

In FPG of islet donors, the negative correlation of cg07814318 methylation was also found with no significance (data not shown). High blood glucose exposure can affect the epigenomic signature of islet cells, differently with obesity pathway. The pancreas should sense blood glucose levels, systemically, and accurately control glucose homeostasis. The epigenetic perturbation of *KLF13* may affect glucose tolerance and obesity separately. However, the obesity related DMP was changed in concordance in circulating blood and target cells. Obesity-related DMP may be surrogate for representing the target cell epigenetic changes by obesity.

Dick *et al*. reported that two SNPs (rs8102595 and rs3826795) of *HIF3A* were independently associated with BMI-related DMP at cg22891070^7^. We also found two *cis-*meQTL SNPs (rs11537749 and rs12595641) of *KLF13* associated with obesity-related cg07814318 methylation. The results of methylation change at cg31624588 by rs11537749 and rs12595641 SNPs remains unclear. Genetic variations located close to the DMPs of *KLF13* might drive the epigenetic change in certain environments through epigenetic regulation of chromatin structure. Therefore, further analysis of meQTL of obesity-related DMPs may explain where and how the sophisticated DNA methylation changes come from. Our data suggest the genetic context of the KLF13 locus could drive epigenetic modification of the obesity-related DMPs in extreme high-BMI children.

## Methods

### Study subjects

Study subjects of this study were selected from the Korean Child-Adolescent Cohort Study (KoCAS), which followed a cohort of Korean students annually at four elementary schools in Gwacheon in 2011–2013^16^. The overall objective of the cohort study was to identify early risk factors for obesity and associated metabolic diseases in Korean children. We defined extreme childhood obesity as a BMI-for-age of ≥1.2 times the 95^th^ percentile value according to the recently proposed definition by the Centers for Disease Control and Prevention. Informed consents were obtained all participants. Adipocyte and Pre-adipocyte are purified from omentum fat tissues, and islets are purified from pancreas after pancreatectomy by following the methods described in the papers^17^. Patient’s consent was acquired, and IRB of Korea National Institute of Health was approved (IRB approved number, 2014-08EXP-05-P-A). Detailed subject criteria are described in Table 1. All experiments for study subjects were performed in accordance with relevant guidelines and regulations.

### Quantitation of CpG DNA methylation by pyrosequencing analyses and Illumina Infinium HumanMethylation450 BeadChip

Polymerase chain reactions (PCRs) of DMPs of *KLF13* were performed for the purpose of pyrosequencing^18^. The PCR cycling conditions and the sequences for all primers designed by MethPrimer (Biotage AB) for cg07814318 and cg31624518 are described in Supplementary Figure 1A. Pyrosequencing assays were designed, optimized, performed on the PSQ HS 96A System (Biotage AB) according to the manufacturer’s specifications (Pyrosequencing, Westborough, MA, USA).

The cg07814318 methylation was determined via the Illumina Infinium HumanMethylation450 BeadChip (Illumin, Inc., San Diego, CA, USA) from 47 human cells, 8 pre-adipocytes, 22 adipocytes purified from abdominal fat tissues and 17 islets from a normal pancreas. Genomic DNA was extracted from each tissue, treated with sodium bisulfite, and subjected to analysis. The methylation levels of CpGs were described as *Beta* values (0 to 1) representing the calculated level of methylation (0% to 100%). The β-value reflects the methylation level of each CpG site. The β-value was calculated by subtracting background using negative controls on the array and taking the ratio of the methylated probe signal intensity to the total locus intensity of both methylated and unmethylated signals. A β-value of 0–1.0 represents a significant proportion of methylation in the range from 0 to 100% for each CpG site. IlluminaBeadstudio v3.1 software (Illumina,Inc., San Diego, CA, USA) was used for quantification and image analysis of methylation data. All methods were performed in accordance with the relevant guidelines and regulations.

### Exome Sequencing

All exons were captured by the SureSelect Human Exon V4 (Agilent Technologies, Carlsbad, CA, USA) according to the manufacturer protocols. The captured DNA was sequenced on the Illumina HiSeq 2500. A 100 bp paired-end exome sequencing was performed using Genome Analyzer (Illumina Inc., San Diego, CA, USA). Base calling was conducted by the Illumina pipeline with the default parameter settings. All sequence data were assembled using the UCSE Genome Browser (GRCh37/hg19) and mapped by BWA (http://bio-bwa.sourceforge.net). Variant detection was performed using SAMTOOLS (http://samtools.sourceforge.net). An average depth of coverage was ~67.2 fold. All methods were performed in accordance with the relevant guidelines and regulations.

### Statistical analyses

For the case-control studies, Welch’s two-sample t-test was used to calculate *P* values. Correlation coefficients between DNA methylation and other traits for linear regression were calculated with Pearson’s test and graphed. Age and gender were used as the covariates. R scripts were used for all other analytical and graphic processing (http://www.r-project.org/). The genome-wide association was analyzed using the PLINK software (http://pngu.mgh.harvard.edu/~purcell/plink).

## Additional Information

**How to cite this article:** Koh, I.-U. *et al*. Obesity-related CpG Methylation (cg07814318) of Kruppel-like Factor-13 (*KLF13*) Gene with Childhood Obesity and its ^cis^-Methylation Quantitative Loci. *Sci. Rep.* **7**, 45368; doi: 10.1038/srep45368 (2017).

**Publisher's note:** Springer Nature remains neutral with regard to jurisdictional claims in published maps and institutional affiliations.

## Supplementary Material

Supplementary Figure and Table

## Figures and Tables

**Table 1:** Summary of the population characteristics for extreme childhood obesity.

Variables	Controls	Cases	*P*
Age	13.93 ± 0.77	13.94 ± 0.84	0.6805
N (M/F)	387 (199/188)	305 (159/146)	0.8784
BMI	19.44 ± 1.34	31.71 ± 3.94	**<0.0001**
WHR	0.76 ± 0.05	0.89 ± 0.06	**<0.0001**
FPG	5.16 ± 0.39	5.23 ± 0.8	**<0.0001**
AST	18.66 ± 3.98	26.33 ± 15.99	**<0.0001**
ALT	11.48 ± 4.05	33.86 ± 33.56	**<0.0001**
TC	156.61 ± 25.62	172.31 ± 31.23	**<0.0001**
TG	70.99 ± 34.62	118.71 ± 66.16	**<0.0001**
HDL	57.18 ± 10.25	46.12 ± 9.01	**<0.0001**

BMI (kg/m^2^): Body Mass Index, FPG (mg/dL): Fasting Plasma Glucose, WHR: Waist to Hip Ratio, AST(IU/L): Aspartate transaminase, ALT(IU/L): alanine aminotransferase, TC(mg/dL): Total Cholesterol, TG(mg/dL): Triglyceride, HDL(mg/dL): High-density lipoproteins -Cholesterol P values < 0.05 are described as bold characters.

**Table 2:** The CpG methylations of KLF13 gene of childhood obesity.

CpG sites	Position	methylation % of Controls (n = 374)	methylation % of Cases (n = 297)	*P*
cg07814318	31624584	**31.2 ± 3.07**	**32.6. ± 3.36**	**2.305e-08**
cg31624588	31624588	32.4 ± 3.27	32.6 ± 3.36	0.385

The values are indicated by mean ± SD, P values < 0.05 are described as bold characters, Position: base positions, OR: Odds Ratio. Welch’s two-sample t-test was used to calculate *P* values.

**Table 3:** The correlation of cg07814318 methylation with glycemic and obesity related traits in KoCAS.

Traits		Total subjects (n = 671)	Control subjects (n = 374)
**BMI**	***Beta***	0.4048	0.0171
(kg/m^2^)	***P***	***1e-06***	*0.492*
**WHR**	***Beta***	0.0051	0.0002
	***P***	***5.801e-14***	***1.196e-14***
**FPG**	***Beta***	−0.1869	−0.2031
(mg/dL)	***P***	***0.0002***	***3.294e-08***
**AST**	***Beta***	0.2456	0.0590
(IU/L)	***P***	***1.495e-08***	***1.279e-11***
**ALT**	***Beta***	0.6433	−0.0060
(IU/L)	***P***	***1.915e-06***	***0.0058***
**TC**	***Beta***	0.2027	−1.1715
(mg/dL)	***P***	***0.0015***	***1.444e-09***
**TG**	***Beta***	0.2971	−1.6185
(mg/dL)	***P***	*0.0743*	***0.0007***
**HDL**	***Beta***	−0.45350	−0.0493
(mg/dL)	***P***	***9.605e-05***	***0.0227***

BMI (kg/m^2^): Body Mass Index, FPG (mg/dL): Fasting Plasma Glucose, WHR: Waist to Hip Ratio, AST(IU/L): Aspartate transaminase, ALT(IU/L): alanine aminotransferase, TC(mg/dL): Total Cholesterol, TG(mg/dL): Triglyceride, HDL(mg/dL): High-density lipoproteins -Cholesterol P values < 0.05 are described as bold characters.

**Table 4:** The association of 9 SNPs of *KLF13* gene with childhood obesity and their correlations with two DMPs.

SNP	BP	Allele	OR	L95	U95	*P*	Functional consequence	cg 07814318	cg 31624588	cis-association (direction)	flanking distance (bp)	R-squared
rs11537749	31619607	A	0.92	0.69	1.22	0.552	synonymous codon	0.0271	0.0007	+	45836	0.142
rs75182225	31619929	A	0.63	0.11	3.46	0.594	missense	0.5968	0.8091	−	45514	NA
rs4779520	31665443	C	0.89	0.71	1.12	0.309	intron variant	0.8657	0.2874	+	0	1
rs117239609	31666335	A	1.20	0.79	1.83	0.388	intron variant	0.4092	0.2195	+	892	0.091
rs6493629	31667796	C	0.88	0.70	1.10	0.256	intron variant	0.8586	0.2532	+	2353	0.93
rs117820946	31668382	A	1.30	0.75	2.24	0.352	intron variant	0.1762	0.6092	+	2939	0.192
rs11537748	31668694	G	1.03	0.83	1.28	0.766	intron variant	0.1504	0.0531	+	3251	0.49
rs12595641	31668706	A	1.09	0.82	1.45	0.559	intron variant	0.0018	0.0167	−	3263	0.109
rs76574759	31669296	A	0.92	0.63	1.35	0.679	intron variant	0.9048	0.6921	+	3853	0.041

**Table 5:** The correlations of each genotype from 2 SNPs with two CpG methylations of DMPs of *KLF13* gene.

SNP	cg	n	*Beta* ± SE	n	*Beta* ± SE	n	*Beta* ± SE	*P*
**rs11537749_A**	**cg07814318**	**480**	31.64 ± 3.15	**145**	32.48 ± 3.57	**23**	31.94 ± 3.98	**0.0271**
	**cg31624588**		32.22 ± 3.13		33.38 ± 3.62		33.15 ± 4.54	**0.0007**
**rs12595641_A**	**cg07814318**	**466**	32.11 ± 3.31	**185**	31.38 ± 3.02	**20**	30.1 ± 3.53	**0.0018**
	**cg31624588**		32.72 ± 3.26		32.11 ± 3.36		31.12 ± 3.55	**0.0167**
