# Supplementary material for: Obesity-related CpG Methylation (cg07814318) of Kruppel-like Factor-13 (KLF13) Gene with Childhood Obesity and its cis-Methylation Quantitative Loci
Source: Sci Rep. 2017 May 16;7:45368. doi: 10.1038/srep45368 (PMC5432839; doi:10.1038/srep45368)
Supplement: Supplementary Figure and Table [file srep45368-s1.pdf]

In-Uk Koh[1€], Hye-Ja Lee[2], Joo-Yeon Hwang[1], Nak-Hyun Choi[1] and Suman Lee[1,*]

Supplementary Fig 1. Pyrogram of two DMPs (cg07814318 and cg31624588) and pyro sequencing conditions

A)

Primer name	Direction	Sequence	Annealing temp./ number of cycles
cg07814318<br>cg31624588	F	5'-biotin-AGGGTTGATAAGTGTTGGTTAA ATG-3'	60 ℃ / 35
	R	5'-CACCACACACTTTCCAATCTCTATA-3'	
	S	5'-AAACCAACCACCAAAC-3'	
	Sequence for analyze	RTACRAAAATAA	

B)

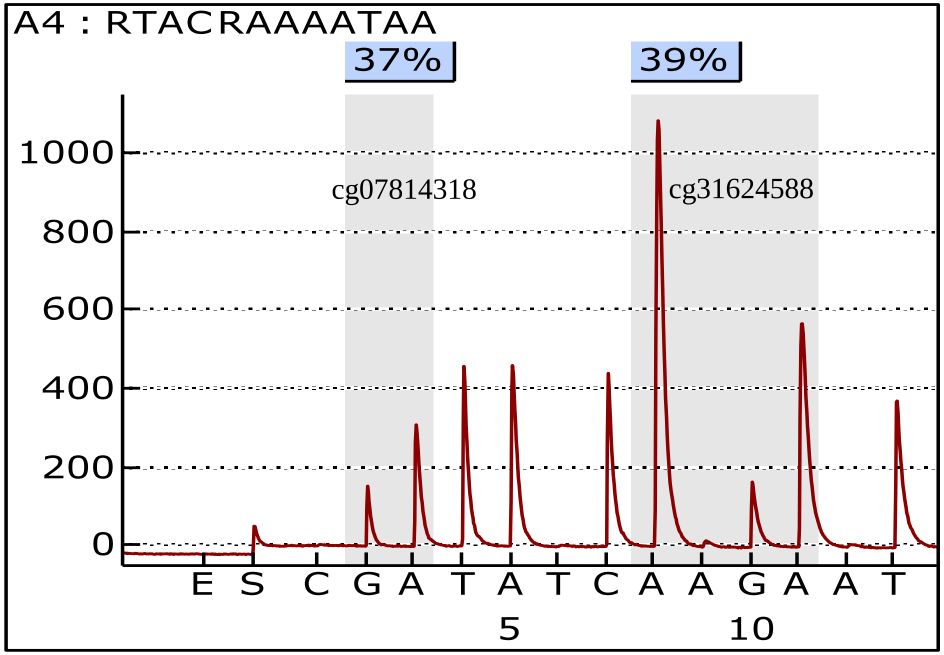

In-Uk Koh[1€], Hye-Ja Lee[2], Joo-Yeon Hwang[1], Nak-Hyun Choi[1] and Suman Lee[1,*]

Supplementary Table 1 . The cg07814318 methylations of *KLF13* gene of human cells

	cg07814318 methylation % of controls (BMI of Donors<29)	cg07814318 methylation % of cases (BMI of Donors>29)	*P*
**Total cells**	77.56652 (n=36)	80.63918 (n=11)	0.0521
**Pre-adipocytes**	76.25 (n=4)	81.39 (n=4)	**0.006**
**Adipocytes**	74.68 (n=18)	74.46 (n=4)	0.9235
**Islets**	79.44 (n=14)	84.08 (n=3)	**0.003**

The values are indicated by mean±SD, *P* values <0.05 are described as bold characters,
Position: base positions, OR:Odds Ratio, Welch's two-sample t-test was used to calculate P values.
